# Bid outcome processing in Vickrey auctions: An ERP study

**DOI:** 10.1111/psyp.14125

**Published:** 2022-06-16

**Authors:** Alice Newton‐Fenner, John Tyson‐Carr, Hannah Roberts, Jessica Henderson, Danielle Hewitt, Adam Byrne, Nicolas Fallon, Yiquan Gu, Olga Gorelkina, Yuxin Xie, Athanasios Pantelous, Timo Giesbrecht, Andrej Stancak

**Affiliations:** ^1^ Department of Psychology University of Liverpool Liverpool UK; ^2^ Institute of Risk and Uncertainty University of Liverpool Liverpool UK; ^3^ Management School University of Liverpool Liverpool UK; ^4^ School of Securities and Futures Southwestern University of Finance and Economics Chengdu China; ^5^ Department of Econometrics and Business Statistics, Monash Business School Monash University Melbourne Victoria Australia; ^6^ Unilever, Research and Development Port Sunlight UK

**Keywords:** ERPs, FRN, P300, reward, Vickrey auction

## Abstract

Online retailers often sell products using a socially competitive second‐price sealed‐bid auction known as a Vickrey auction (VA), an incentivized demand‐revealing mechanism used to elicit players' subjective values. The VA presents a situation of risky decision‐making, which typically implements value processing and a loss aversion mechanism. Neural outcome processing of VA bids are not known; this study explores this for the first time using EEG. Twenty‐eight healthy participants bid on household items against an anonymous, computerized opponent. Bid outcome event‐related potentials were predicted to differentiate between three conditions: outbid (no‐win), large margin win (bargain), and small margin win (snatch). Individual loss aversion values were evaluated in a separate behavioral experiment offering gains or losses of variable amounts but equal chances against an assured gain. Processing outcomes of VA bids were associated with a feedback‐related negativity (FRN) potential with a spatial maximum at the vertex (251–271 ms), where bargain win trials resulted in greater FRN amplitudes than snatch win trials. Additionally, a P300 potential was sensitive to win versus no‐win outcomes and to retail price. Individual loss aversion level did not correlate with the strength of FRN or P300. Results show that outcome processing in a VA is associated with FRN that differentiates between relatively advantageous and less advantageous gains, and a P300 that distinguishes between the more and less expensive auction items. Our findings pave the way to an objective exploration of economic decision‐making and purchasing behavior involving a widely popular auction.

## Abstract 2

This study is the first to utilize a prominent online purchasing paradigm to provide insight into the neural mechanisms of economic decision‐making under risk. Feedback‐related potentials showed sensitivity to instantaneous evaluations of the relative value of gain outcomes, and encoded the magnitude of the auctions outcome rewards.

## 1 INTRODUCTION

As electronic commerce continues to dominate retail markets, it is vital to understand decision‐making in online purchasing contexts (Cinar, 2020; Nguyen et al., 2018; Rose et al., 2011). In value‐based decision‐making research, subjective valuations are often quantified in the form of willingness‐to‐pay (WTP), where a person assigns a monetary unit to the value of obtaining a good or experience. This has the advantage that valuations within and across domains (such as food, pain, people, and experiences) can be compared on the linear scale of a given currency. Auction paradigms are widely used to quantify WTP in neuroeconomics research; the most well‐established of these being the Becker‐DeGroot‐Marschak (BDM) auction (Becker et al., 1964; Peters & Buchel, 2010; Plassmann et al., 2007; Roberts et al., 2018, 2022; Tyson‐Carr et al., 2018, 2020). Further, several multinational auction websites utilize a format that is strategically equivalent to the BDM: the Vickrey auction (VA) (Barrot et al., 2010).

The VA and BDM share the same basic paradigm: players put forward a single bid privately, the highest bidder wins and pays the value of the second highest bid. All other players win nothing and lose nothing. In both auctions, the game‐theory dominant strategy, that is, the best reply to every strategy profile of all other players, is to bid the maximum amount one is willing to pay (Vickrey, 1961). Therefore, the VA and BDM allows for the inference of the participant's subjective values of items while manipulating the behavior of their opponent and therefore the auction outcomes (Noussair et al., 2004).

The VA and BDM are both demand‐revealing mechanisms, but differ in two major respects: the identity of the bidder's opponent(s) and the amount of outcome feedback (Noussair et al., 2004). In a BDM, the player bids against a random number generator and is told whether they won or lost; whereas in a VA the players are aware of competing with other anonymous, human players, and the winner is also told the final price paid. This price is wholly dependent on the bid of the losing player (or in the case of multiple opponents the second highest bidder) and therefore, the winner receives information about their opponents' subjective values, and whether their values align.

Furthermore, by revealing the final price paid in winning trials, wins can be divided into more‐ and less‐advantageous outcomes. If there is a large difference between the bids, the winner will pay significantly less than they were willing; an outcome hence referred to as a bargain trial. Meanwhile, if both players place similar bids, the final price paid will be much closer to the winner's WTP; an outcome hence referred to as a snatch trial. Both outcomes are considered “good” as the participant pays less than their WTP, but the margin of difference between their bid and the final price paid can be controlled. This is a unique advantage of the VA, as it allows for intermediate outcomes on a scale of relative good–bad in the win domain, so that some wins can be more extreme than others. Previous behavioral studies of economic decision‐making have also demonstrated that subjective value is sensitive to social factors, such as how one's performance fairs against others (Fehr & Schmidt, 1999). Therefore, decision‐making in the VA could employ different reward processing mechanisms to the BDM, by virtue of social context information and the competitive environment (Chen, 2011; Malhotra & Bazerman, 2008; van den Bos et al., 2013).

In EEG research, the most prominently investigated event‐related component (ERP) connected to outcome evaluation in decision‐making tasks involving uncertainty is the feedback‐related negativity (FRN) (Walsh & Anderson, 2012). Also referred to as the feedback error‐related negativity, (fERN) (Holroyd & Coles, 2002), the medial frontal negativity (MFN) (Gehring & Willoughby, 2002), feedback negativity (FN) (Hajcak et al., 2006), and most recently the reward positivity (RewP) (Proudfit, 2015), it is a suppressed or otherwise obliterated negative deflection elicited by win outcomes approximately 200–300 ms post feedback‐onset, which is not present in loss outcomes. It is typically measured from a single electrode in the midline frontal‐central area (Glazer et al., 2018), and has been posited to reflect a reinforcement learning reward prediction error (Holroyd & Coles, 2002), consistently differentiating between context‐dependent favorable and unfavorable outcomes (Hajcak et al., 2006; Holroyd et al., 2004). It was initially theorized to reflect a subjective “worse than expected” error signal (Hajcak et al., 2007; Nieuwenhuis et al., 2004). However, current research suggests that the apparent negativity of the FRN waveform is produced by a conflation of the N200 potential with the RewP component, where all outcome feedback elicits an N200, but a RewP suppresses this N200 in gain outcomes in this time range (Holroyd et al., 2008; Proudfit, 2015). Here, we define the FRN as the difference waveform between averaged potentials time‐locked to win and no‐win outcome‐feedback. As VA involves outcome uncertainty, it is likely that feedback processing of auction outcomes entails an FRN.

Additionally, the P300 event‐related component—in particular the P3b sub‐component—is also thought to be involved in outcome evaluation. The P3b has a positive‐going amplitude which occurs approximately 300–500 ms after stimulus onset, and typically peaks at its maximum amplitude at parietal electrode sites, most commonly Pz, CPz, and Cz (Polich, 2007, 2012). While the P300 is typically produced by non‐frequent target stimuli interspersed among frequent standard stimuli (Duncan‐Johnson & Donchin, 1977; Polich, 2012; Polich & Margala, 1997), it also has well established sensitivities to outcome magnitude, particularly in purchasing and social contexts (Bellebaum et al., 2010; Jones et al., 2012; Pfabigan & Han, 2019; San Martin, 2012; Schaefer et al., 2016; Yeung & Sanfey, 2004). Recent literature has also shown that the P300 is modulated by social feedback in economic contexts (Mussel et al., 2022; Weiß et al., 2020), with a larger positivity for positive and unexpected feedback. The P300 is also modulated by outcome probability, with studies utilizing gambling paradigms demonstrating that unexpected rewards elicit stronger P300 amplitudes compared to expected rewards (Cohen et al., 2007; Hajcak et al., 2005, 2007). Taken together, we postulated that a series of VA trials would elicit a P300 potential, differentiating between win and no‐win outcomes.

Research into reward processing typically investigates decision‐making in conditions of risk and uncertainty, most commonly using a variant of a gambling task (Chandrakumar et al., 2018). These scenarios also often engage loss aversion: a greater sensitivity to potential losses than potential gains (Kahneman & Tversky, 2013). For example, loss aversion is correlated with greater autonomic responses to losses (Sokol‐Hessner et al., 2013; Stancak et al., 2015) and stronger activation in the amygdala in the outcome period during gambling tasks (Canessa et al., 2013; Sokol‐Hessner et al., 2013). Most relevantly, Kokmotou et al. (2017) found a positive correlation between loss aversion and FRN amplitude in the outcome evaluation period of a monetary gambling task. Most gambling tasks have known probabilities of outcomes (e.g. a 50:50 gamble) whereby participants can quantify the static risk level and behave accordingly (Kokmotou et al., 2017). During a VA, by using an anonymous opponent with an unknown strategy, the players are put into a situation of unpredictable uncertainty. This allows investigation of the role of uncertainty in decision‐making as a separate entity to risk. A secondary aim of the current study was to investigate loss aversion implementation in this real online purchasing scenario.

To date, no studies have explored the neural mechanisms implemented during a VA, despite its widespread use in online retail. Unlike other decision‐making tasks under uncertainty, no outcome in a VA can be classified as a financial loss, and so it is unclear whether processing outcomes in a VA would be associated with FRN and a P300. This study examined for the first time the FRN and P300 components of ERPs elicited by receiving outcomes of bids in a VA, and explored the nuance of ERP responses to different types of wins in a win versus no‐win context (e.g. high retail value wins vs. low retail value wins, and bargain wins vs. snatch wins). It was hypothesized that processing outcomes in a VA will be accompanied by the FRN and P300 ERP components. Further, we predicted that the relatively advantageous win outcomes (bargain) will show greater FRN than the relatively disadvantageous (snatch) win outcomes. Finally, given the presence of FRN in the data, we also postulated a positive association between individual loss aversion levels and the strength of FRN.

## 2 METHOD

### 2.1 Participants

Twenty‐eight healthy participants (12 male, 25 right handed) with a mean age of 25.9 ± 6.9 years (mean ± SD), took part in the current study. Three participants (two male) were removed from subsequent analyses due to excessive muscle artifacts in EEG recordings. One participant (male) was excluded due to not bidding in 65% of trials. All had normal or corrected‐to‐normal vision. All participants were screened for psychological/psychiatric disorders. A post hoc sensitivity analysis confirmed that the one‐way within‐subjects ANOVA with 24 participants across three outcome conditions would be sensitive to effects of *ƞ*_p_^2^ = 0.21 with 80% power (*α* = .05). The experimental procedures were approved by the Research Ethics Committee of the University of Liverpool. All participants gave written informed consent in accordance with the Declaration of Helsinki. Participants were reimbursed for their time and travel expenses.

### 2.2 Procedure

The study was carried out in a single session. Participants completed an EEG experiment involving a computerized VA task, and a behavioral computerized monetary gambling task to measure loss aversion. The purpose of the experiment and instructions for the tasks were explained to participants at the beginning of the session. All experimental procedures were carried out in a dimly lit, sound‐attenuated Faraday cage. Both tasks were displayed on a 19‐inch LED monitor using MATLAB (Mathworks, Inc., USA), with Cogent software 2000 (Cogent, www.vislab.ucl.ac.uk/Cogent/).

#### 2.2.1 VA task

Participants received an initial endowment of £18 and were instructed to use it to purchase items during the VA task. They were informed that two items from winning trials would be randomly selected and the price that they won the items for would be deducted from their endowment; they would receive the remaining amount of their endowment and the two items as reimbursement for their participation. After application of the EEG net, participants were led into the Faraday cage to complete the task. Participants were seated in front of the computer and rested their dominant hand on a computer mouse.

**FIGURE 1:**
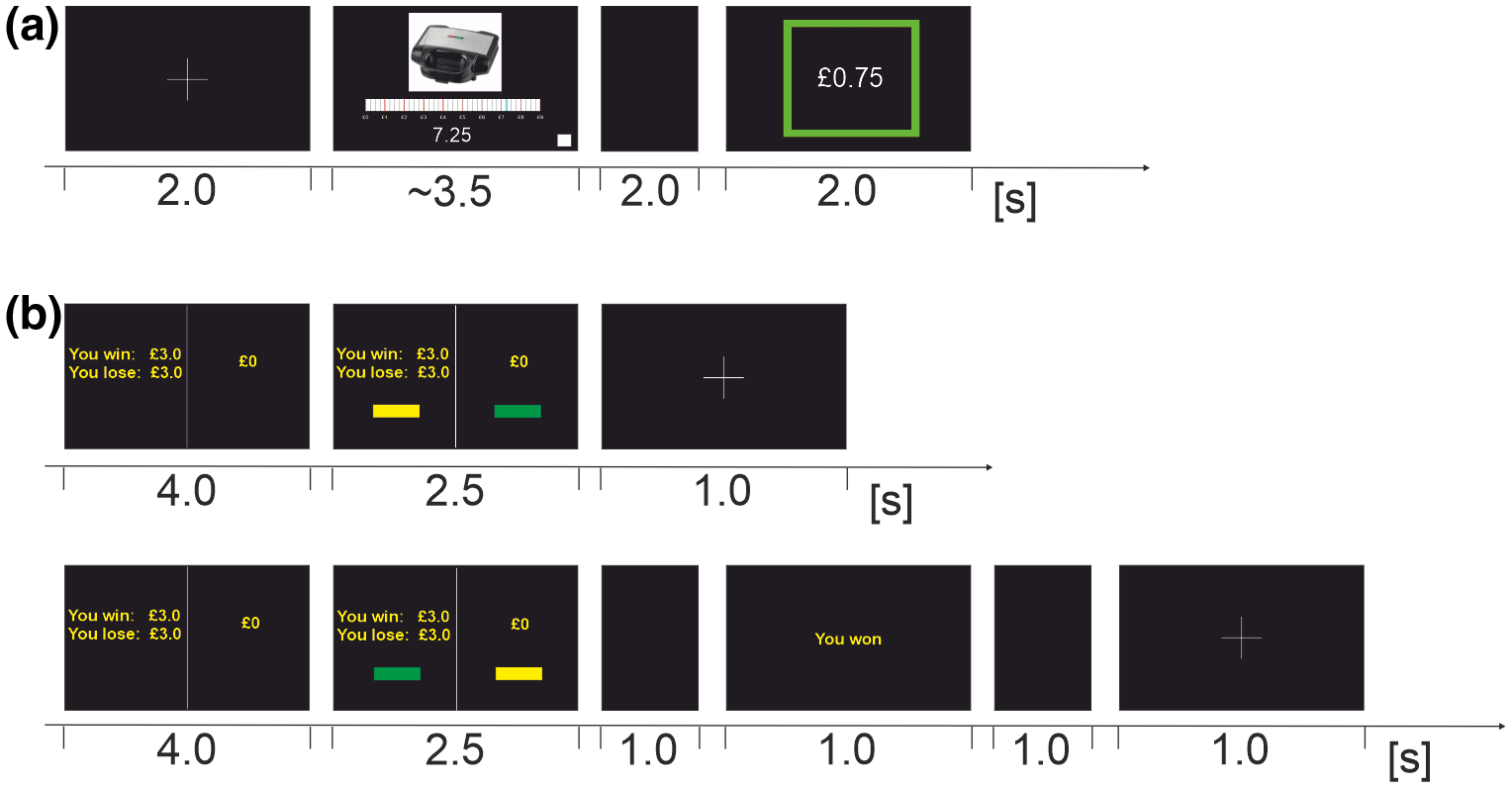
(a) Trial structure of Vickrey auction task in the bargain condition. Each trial began with a fixation cross for 2 s, followed by the auction item and a sliding scale from £0–£9 in increments of 25p on which to select their bid. Participants were instructed to select their bid on the scale, and once they were happy with their decision submit the bid by clicking on a white square in the bottom right‐hand corner. The screen was blank for 2 s before presenting the outcome of the trial. If the participant won the trial (presented) a green square appeared with the amount they won the item for in the center of the square. If the participant lost the trial, 25p more than their bid value was shown at the center of a red square. (b) Trial structure of loss aversion task. Top panel: Declined gambles. Bottom panel: Accepted gambles. Each trial began with a fixation cross, followed by the presentation of two possible choices, which were displayed on the screen for 4 s. Half of the screen showed the gamble option (e.g. “you win £3.0, you lose £3.0”) with a 50:50 chance of winning or losing the displayed amount of money. The other half of the screen showed the value of a sure outcome. In the next 2.5 s, the options stayed on the screen and two yellow rectangles appeared at the bottom of the screen. Participants were instructed to choose between the two options by pressing the left or the right mouse button corresponding to the side of the screen they preferred. If the participants selected the sure outcome, a fixation cross appeared on the screen and the next trial started after 1 s. If participants selected the risky gamble option, a black screen was displayed for 1 s after the 2.5 s response period, and feedback about the gamble outcome was shown for 1 s (“You won” or “You lost”). A 1 s black screen served as a resting period before the next trial.

The protocol for the VA task was adapted from previous studies (Kokmotou et al., 2017; Roberts et al., 2018; Tyson‐Carr et al., 2018, 2020) which used a BDM paradigm. The trial structure is shown in Figure 1a. The order of item presentation was randomized between participants, and each item was presented once, resulting in a total of 300 auction trials. The stimuli comprised 300 everyday household products such as kettles, batteries, and mugs, valued in the ranges £3–5 (low value) and £7–9 (high value; *n* = 150 in each range), with a mean value of £6.04 ± £2.19 (mean ± SD) obtained from a shopping catalogue. The items were chosen for their ubiquity, utility, and price point, as we wanted the participants to be familiar with the type of items they were bidding on and view them as desirable. Each auction trial began with a resting interval during which participants viewed a white fixation cross on a black background for 2 s. The participants were then presented with an item to bid on, using a sliding scale from £0–£9 in increments of 25p, giving a total of 37 options. Participants were asked to bid the maximum amount they would be willing to pay for each item, and to select their bid value by clicking on the scale, and submit the bid by clicking on a white square in the bottom right‐hand corner. There was no time limit on bid submission and participants could click on the scale as many times as they wished before submitting the bid.

After bid submission, the trial outcome was determined randomly by the computer, with three equally likely outcomes: (1) the participant is outbid (no‐win condition); (2) the participant has won by a small margin, paying 70%–90% of the value of their bid (“snatch” condition); (3) the participant has won by a large margin, paying 10%–30% of the value of their bid (“bargain” condition). In outcome (1), the value of the participant's bid plus a 25p increment appeared in the center of a red square for 2 s. In outcome (2) and (3), prices were rounded to the nearest 25p and displayed at the center of a green square for 2 s.

The task consisted of a total of 300 trials, split into three blocks of 100 trials each. Trials were presented in random order for each participant. Participants were given a short break in between blocks to limit fatigue. The duration of each block was approximately 15 min. After the VA task was complete, participants were given a short break and the EEG system was removed.

#### 2.2.2 Loss aversion task

The loss aversion task was adapted from previous studies (Kokmotou et al., 2017; Stancak et al., 2015). Participants were given £10 as an initial endowment to use during the task. They were told that 10% of the final amount of money gained or lost during the task would be added to or subtracted from the endowment, and the remaining amount would be given as compensation for their travel costs and time.

The task consisted of 100 two‐alternative forced‐choice monetary gamble trials. In 80 of those trials, participants chose between a 50:50 gamble and a sure zero outcome. The gamble options comprised of eight possible gain amounts (£1.00, £2.00, £3.00, £3.50, £4.50, £5.00, £5.50, £6.00) and 10 possible loss amounts, which were devised by multiplying the given gain value with a number between 0.2–2.0 in 0.2 increments. All possible permutations were presented in the task trials (8 gains × 10 losses). In the other 20 trials, participants chose between a gain‐only gamble and a sure smaller gain. In these trials, the gain‐only gambles presented a 50:50 chance to win a certain gain amount or a zero outcome. The list of assured gains was identical to our previous study (Stancak et al., 2015). Trial order was randomized for each participant.

The trial structure of the loss aversion task can be seen in Figure 1b. At the beginning of each trial a fixation cross appeared on screen for 1 s, followed by the two alternative options for 4 s. One side of the screen showed the gamble option (e.g. “you win £2.0, you lose £1.5”), and the other side of the screen showed the sure outcome option. Participants were told that the gamble option had a 50:50 probability of winning or losing. They were told to make their selection by pressing the left or right mouse button as it corresponded to their choice on the monitor. When the participant chose the gamble option, outcome feedback appeared on screen for 1 s (“you won” or “you lost”). The task took approximately 10 min to complete.

### 2.3 EEG recordings

EEG was recorded continuously using a 129‐channel Geodesics EGI System (Electrical Geodesics, Inc., Eugene, Oregon, USA) with a sponge‐based HydroCel Sensor Net. This system allows full head electrode coverage as it includes electrodes positioned over lower scalp regions and face, which is essential for identification of deep cortical sources, such as those located in orbitofrontal cortex (Luu et al., 2001, 2009; Sperli et al., 2006; Tucker, 1993). The sensor net was aligned with respect to three anatomical landmarks: two preauricular points and the nasion. Electrode‐to‐skin impedances were kept below 50 kΩ and at equal levels across all electrodes, as recommended for the system (Ferree et al., 2001; Luu et al., 2003; Picton et al., 2000). The recording band‐pass filter was 0.001–200 Hz with sampling rate of 1000 Hz. The electrode Cz served as the reference electrode.

### 2.4 ERP analysis

The ERP analysis of the outcome period served to evaluate the individual feedback‐related potentials FRN and P300. EEG data were preprocessed with the BESA v. 7.0 program (MEGIS, Munich, Germany). EEG signals were spatially transformed to reference‐free data using common average reference method (Lehmann et al., 1987). This spatial transformation restored the signal at electrode Cz for use in further analyses.

During preprocessing, EEG data were filtered (0.5–70 Hz with a 50 Hz notch filter) for viewing both slow‐frequency, for example, movement or pressure pulse, and high‐frequency, for example, EMG, artifacts. Ocular artifacts and, when necessary, electrocardiographic artifacts were removed with principal component analysis based on averaged artifact topographies (Berg & Scherg, 1994; Ille et al., 2002). Data were also visually inspected for the presence of atypical electrode artifacts. In rare cases where an electrode signal was continually affected by artifacts, the electrode signal was interpolated. Continuous data were sectioned into epochs of 900 ms duration each with a baseline interval ranging from −300 to 0 ms relative to feedback‐onset.

The average number of accepted trials in each condition were: no‐win, 96.8 ± 15.8 (mean ± SD); bargain win, 89.9 ± 11.4; snatch win, 85.0 ± 10.0. Paired *t* tests revealed that the average number of accepted trials differed between the snatch win and other conditions (*p* < .05) but did not differ between no‐win and bargain win conditions (*p* > .05), or between number of accepted trials in low‐value and high‐value conditions (low = 135.3 ± 9.4, high = 136.4 ± 9.1; *p* > .05). Data were filtered from 0.5–30 Hz. ERPs in response to outcome feedback were computed separately for each condition by averaging respective epochs in the intervals ranging from −300 to 600 ms post feedback‐onset. The FRN potential was quantified by subtracting ERPs of no‐win trials from ERPs of bargain/snatch trials (analogous to a win‐minus‐loss difference waveform).

In the VA task, EEG epochs were averaged for each type of outcome (snatch, bargain, and no‐win) and for both market value categories (high and low). Based on visual inspection of scalp topographies and previous research (Glazer et al., 2018; Hauser et al., 2014; Krigolson, 2018; Meadows et al., 2016; Walsh & Anderson, 2012), the Cz electrode was selected for statistical analysis. Intervals of interest were selected based on visual inspection and a permutation test involving 4000 permutations and implemented in the *statcond.m* function of the EEGLAB toolbox (Delorme & Makeig, 2004; Maris & Oostenveld, 2007). The time windows of interest chosen were 251–271 ms (FRN) and 354–374 ms (P300) post feedback‐onset. Graphical representations of these intervals can be seen in as gray bars in Figure 3b,c for FRN, and 4b for P300.

### 2.5 Statistical analysis

#### 2.5.1 Behavioral data

For the VA task, trials in which the participant did not bid were excluded due to lack of engagement in the trial and the resulting outcome. Response times were uninformative as judgments were not time limited. A one‐way repeated measures ANOVA was conducted to examine the effect of market value category on bid value.

As outcome probabilities were fixed, the participant was pre‐determined to win two thirds of the trials. While participants were instructed that the dominant strategy was to bid one's true subjective value, the true dominant strategy was to bid the smallest amount possible: 25p per trial. In order to test for any implicit learning during the task, we conducted a Pearson's correlation between the trial number and bid value to test for a general trend of lowering of bids as the task progressed.

For the loss aversion gambling task, Shapiro–Wilk tests were conducted to confirm normal distributions across loss aversion, risk aversion, and choice sensitivity parameters.

#### 2.5.2 ERP data

For the FRN, in line with previous studies (Chandrakumar et al., 2018; Glazer et al., 2018; Walsh & Anderson, 2012), win trials were subtracted from no‐win trials in order to establish the difference waveform, and to select the appropriate electrode and latency epoch showing a statistically significant effect. A one‐way repeated measures ANOVA was conducted examining outcome condition (no‐win, bargain, and snatch), and a subsequent 2 × 3 repeated measures ANOVA was conducted comparing the effects of value (high vs. low) and outcome condition (no‐win, bargain, and snatch) on ERP amplitudes.

For the P300, four electrodes of interest corresponding to Fz, FCz, Cz, and Pz in the 10–20 electrode system, numbered 11, 6, 129, and 62, respectively in the HydroCel Geodesic net, were selected to account for the whole positive maximum of the P300 potential. A 4 × 2 × 3 repeated measures ANOVA with factors of electrodes (four electrodes), value (low vs. high), and bid outcome conditions (bargain, snatch, and no‐win) was carried out. A subsequent 2 × 3 (value × outcome) repeated measures ANOVA was carried out to unpack the relationship between outcome condition and item market value in electrode 6.

In both components, Greenhouse–Geisser corrections were utilized whenever the sphericity assumption was violated. Significant differences outlined in the ANOVA were subjected to pairwise *t* tests with Bonferroni corrections and a critical threshold of *p* < .05 was upheld. A 95% confidence level was always employed.

## 3 RESULTS

### 3.1 Behavioral data

#### 3.1.1 The VA task

Participants submitted bids in 94.2% of trials. The maximum bid of £9 was submitted on 2.9% of trials. The overall mean bid value was £3.36 (SD ± 2.5), £2.59 less than the mean market value of the items. Average bid value rose slightly as the task progressed: *r*(22) = .63, *p* < .001.

**FIGURE 2:**
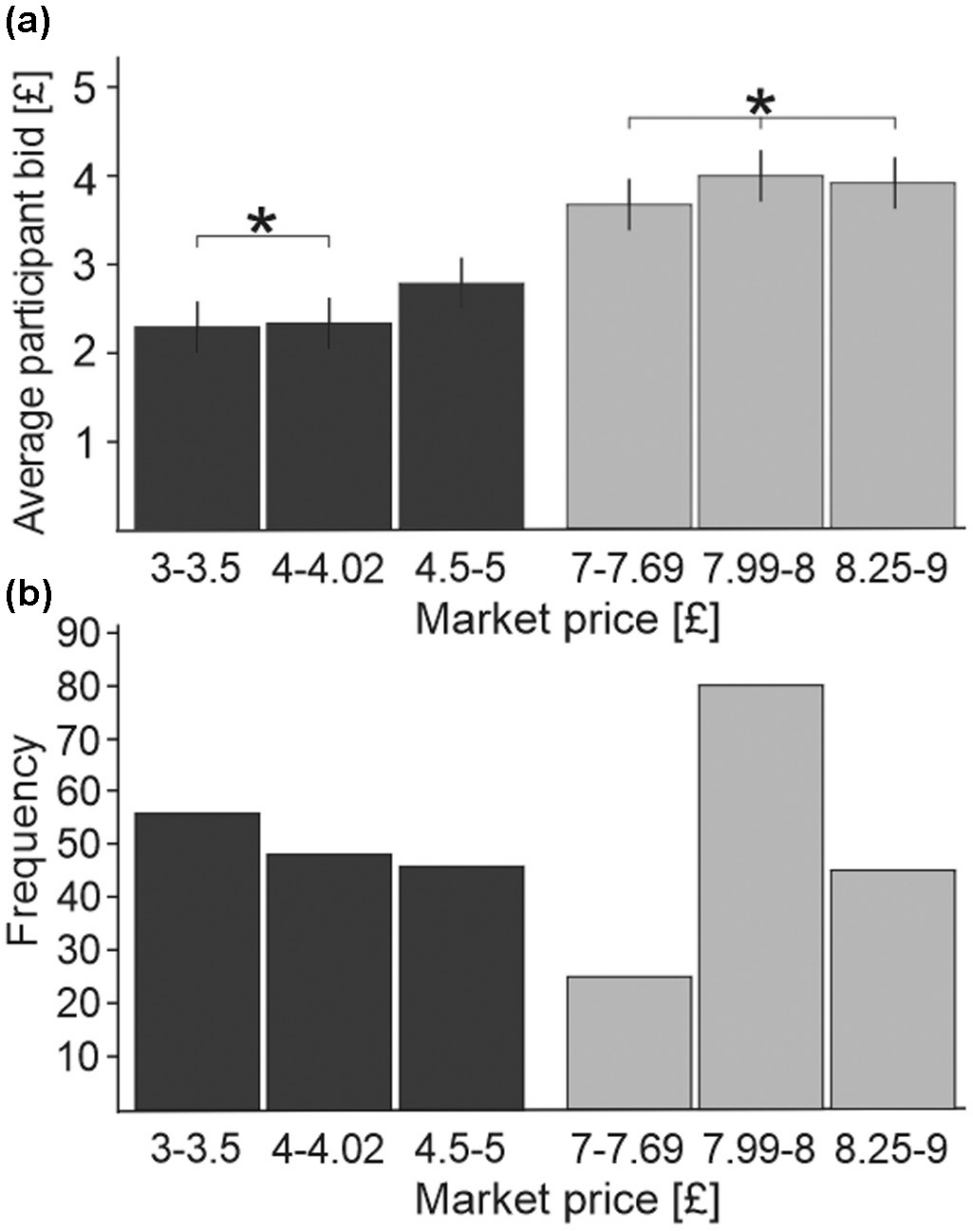
(a) Bar graph showing mean participant bids in the VA task across six levels of market value: three subsections of low value (£3–5, dark gray) and high value (£7–9, light gray). The subsections were grouped according to frequency of price, as seen in (b). All levels of participants' bid value differed between the six levels of market value except for the brackets highlighted by a *. (b) Bar graph showing the frequencies of market prices among 300 auction item stimuli corresponding to the six levels of market value in (a). Efforts were made to distribute prices evenly within the high and low value ranges.

Figure 2a shows a statistically significant relationship between participants' mean bid value and the six levels of market value (*F*(5,115) = 68.11, *p* < .001, *ƞ*_p_^2^ = .75). Post hoc pairwise comparisons with Bonferroni corrections showed differences across all value levels (*p* < .05) except between the £3–3.5 and £4–4.02 brackets, and between the three high market value brackets (*p* > .05). The participants were not told the retail price of the auction items so as not to anchor their bids, but the significant relationship between participant bid value and market value validates the use of market value as a proxy measure in the analysis. Additionally, there was a highly significant linear trend (*p* < .001), confirming a linear increase in subjective value with increase in retail price. This suggests that subjective value ratings within the VA reflects the retail prices of the products. The distribution of market price frequencies among the 300 auction item stimuli can be seen in Figure 2b.

#### 3.1.2 Loss aversion task—Choice parameters

Loss aversion (*W*(23) = .98, *p* > .05), risk aversion (*W*(23) = .97, *p* > .05), and choice sensitivity were all normally distributed (*W*(23) = .96, *p* > .05). The mean level of loss aversion (*λ*) was 1.38 ± 0.10 (mean ± SEM). This value fit well with previous studies of *λ* = 1.4 (Sokol‐Hessner et al., 2009; Stancak et al., 2015). There was no correlation between loss aversion and risk aversion (*p* > .05).

### 3.2 ERP results

#### 3.2.1 FRN

**FIGURE 3:**
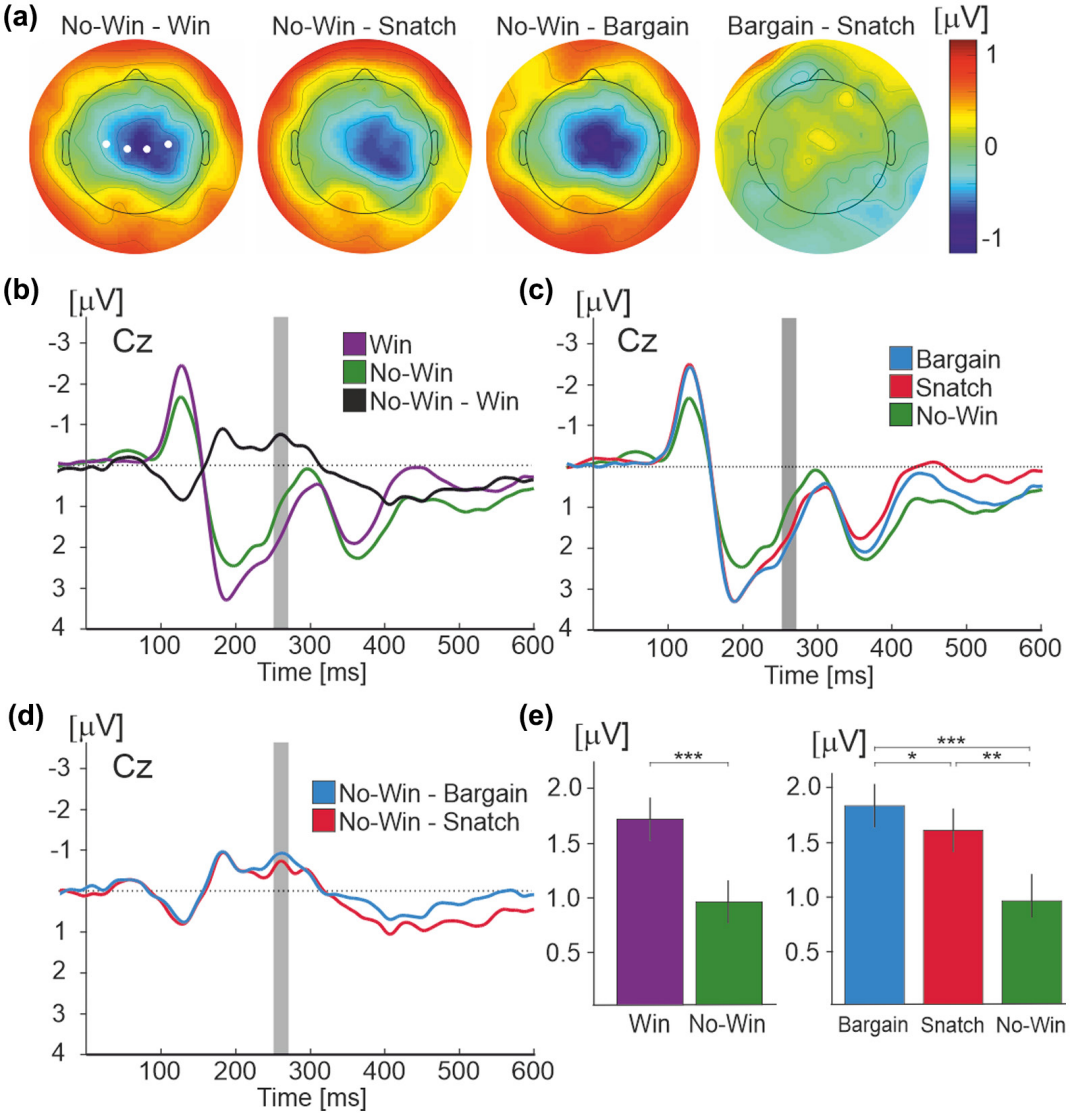
FRN component. (a) Whole scalp topographic maps displaying differences in grand average ERPs at time point (251–271 ms). (b) Grand average ERP waveform across all participants and product value conditions comparing win (purple), no‐win (green) outcome conditions, and the no‐win minus win difference waveform (black) at electrode Cz. Epoch of interest showing statistically significant differences between win and no‐win conditions (251–271 ms post feedback‐onset) highlighted in gray. (c) Grand average ERP waveform across all participants and product value conditions comparing the no‐win outcome condition (green) to the two types of win condition — bargain (blue) and snatch (pink) at electrode Cz. Epoch of interest (251–271 ms post feedback‐onset) is highlighted in gray. (d) Grand average difference ERP waveform across all participants and product value conditions comparing the no‐win minus bargain win (blue) and the no‐win minus snatch win (pink) at electrode Cz. Epoch of interest (251–271 ms post feedback‐onset) is highlighted in gray. (e) Bar graphs showing mean amplitude of ERPs over epoch 251–271 ms for (b) and (c). Statistically significant differences are denoted as * for <.05, ** for <.01, and *** for <.001. The error bars show the standard error.

An FRN with a spatial maximum at the central midline electrode Cz was found in response to bidding outcomes in VA during the epoch 251–271 ms (Figure 3a).

From visual inspection of the topographic plots, the FRN appeared to be stronger in the right hemisphere, as can be seen on the topographic maps in Figure 3a. To verify a right lateralization effect, a repeated measures ANOVA was conducted comparing activity at the Cz electrode with the electrodes on the right and left of Cz (electrodes 36, 31, 80, and 104 were selected). No significant difference was found between electrodes (*p* > .05).

The grand‐average ERP waveforms at electrode Cz for win and no‐win conditions are shown in Figure 3b. Figure 3c,d demonstrate a main effect of condition (*F*(2,46) = 16.90, *p* < .001, *ƞ*_p_^2^ = .42). Significant differences were found between all three outcomes, with bargain trials (1.84 ± .38 μV) resulting in more positive potential amplitudes than snatch trials (1.61 ± .39 μV, *p* = .036), and both bargain and snatch trials resulting in more positive amplitudes than no‐win trials (0.95 ± .38 μV, *p* < .001 and *p* = .001, respectively). The subsequent 2 × 3 ANOVA found no statistically significant main effect of value or interaction between values and condition (*p* > .05).

#### 3.2.2 P300 component

**FIGURE 4:**
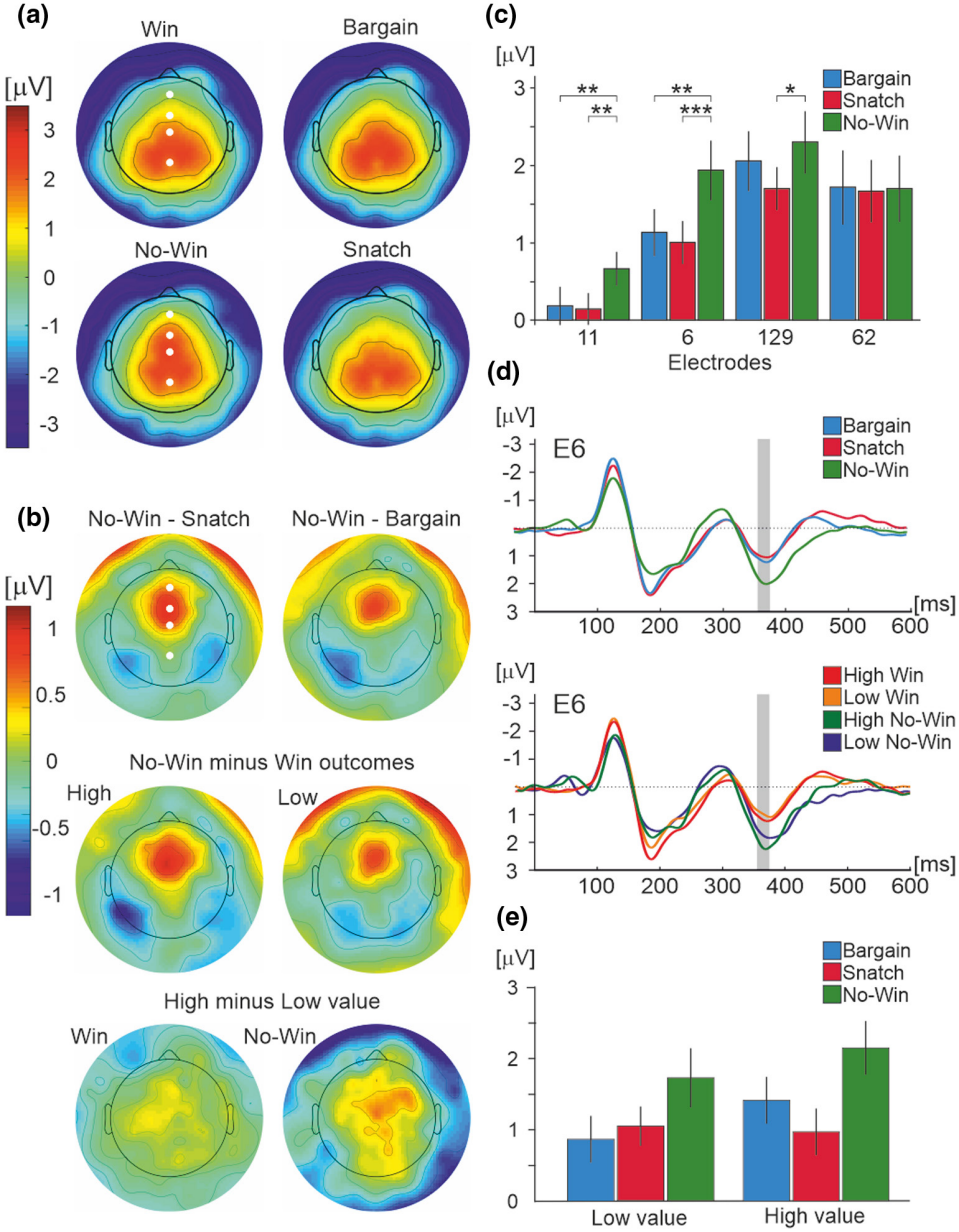
P300 component. (a) Whole scalp topographic maps displaying grand average ERPs for each of the outcome conditions at time point 354–374 ms. Four midline electrodes used in statistical analysis, numbered 11 (Fz in 10–20 system), 6 (FCz), 129 (Cz), and 62 (Pz) in HydroCel Geodesic net, are highlighted in white. (b) Whole scalp topographic maps displaying difference in grand average ERPs between conditions in the latency epoch of 354–374 ms. (c) Bar graph showing mean amplitude of ERPs over epoch 354–374 ms for all outcome conditions across four midline electrodes 11, 6, 129, and 62, as indicated by white circles on topographic maps in (a) and (b). The error bars show the standard error. Statistically significant differences in the bar graphs are denoted as * for <.05, ** for <.01, and *** for <.001. (d) Top: Grand average ERP waveform across all participants and product value conditions comparing outcome conditions at electrode 6. Bottom: Grand average ERP waveform across all participants comparing no‐win outcomes with high (green) and low (blue) market value to win outcomes with high (red) and low (orange) market value at electrode 6. Epoch of interest 354–374 ms post feedback‐onset is highlighted in gray. (e) Bar graph showing mean amplitude of ERPs over epoch 354–374 ms for all outcomes and market values.

Topography of the P300, as can be seen in Figure 4a, showed bilateral positivity over the parietal electrodes, peaking at 354–374 ms. The topographic maps of the P300 component in win and no‐win conditions showed spatial maximum at central parietal locations, and the greatest differences between conditions were maximal at midline frontal‐central electrodes.

A main effect of electrodes (*F*(3,69) = 6.69, *p* = .004, *ƞ*_p_^2^ = .225), value (*F*(1,23) = 8.81, *p* = .007, *ƞ*_p_^2^ = .277), and outcome conditions (*F*(2,46) = 7.89, *p* = .001, *ƞ*_p_^2^ = .255), and a statistically significant interaction between electrodes and bid outcome conditions (*F*(6,138) = 4.19, *p* = .006, *ƞ*_p_^2^ = .154) was found. The main effect of value was due to the larger P300 potential in high‐ compared to low‐value items (high: 1.48 ± .24 μV; low: 1.23 ± .21 μV, mean ± SEM).

Subsequent analysis revealed that the main effect of electrodes was due to significant differences in amplitudes between all electrodes apart from between 62 and 11, and between 129 and 62 (*F*(3,69) = 6.61, *p* = .001, *ƞ*_p_^2^ = .223). A stronger positive P300 potential was observed at the electrodes located at vertex (electrode 129: 2.01 ± .35 μV) and in the parietal scalp region (electrode 62: 1.67 ± .43 μV) compared to two electrodes located anteriorly relative to the vertex electrode (electrodes 11: .34 μV ± .20 μV, and 6: 1.37 ± .30 μV; see Figure 4c). Figure 4b shows topographical maps of the difference in potential amplitude of the P300 between conditions. Notably, the topographic maps of the contrast no‐win versus both win conditions revealed that only the anterior part of the P300 potential maximum, represented in electrodes 11 and 6, resolved the bid outcome conditions.

The main effect of bid outcome conditions (*F*(2,46) = 7.59, *p* = .001, *ƞ*_p_^2^ = .248) was related to a stronger P300 in no‐win outcomes (1.64 ± 0.27 μV, mean ± SEM) compared to both bargain (1.28 ± 0.25 μV) and snatch (1.12 ± 0.19 μV) outcomes; the two win outcomes did not significantly differ. The interaction between electrodes and bid outcomes (Figure 4d) revealed that the amplitudes differed between no‐win and both win outcomes in electrodes 11 (bargain: .18 ± .24 μV, *p* = .009; snatch: .16 ± .20 μV, *p* = .001; no‐win: .66 ± .22 μV,), and six (bargain: 1.15 ± .31 μV, *p* = .001; snatch: 1.01 ± .27 μV, *p* < .001; no‐win: 1.94 ± .39 μV), at electrode 129 no‐win differed from snatch but not from bargain (bargain: 2.05 ± .40 μV *p* > .05; snatch: 1.69 ± 2.8 μV *p* = .012; no‐win 2.29 ± .41 μV), and the outcomes did not differ at all in the parietal electrode 62 (*ps* > .05), in accordance with the topographic maps of bid outcome contrasts (Figure 4b). This can be seen in Figure 4d.

Figure 4e shows the grand mean ERP amplitudes at electrode 6 in each of three bid outcomes and for high‐ and low‐value items, as evaluated in the subsequent 2 × 3 repeated measures ANOVA. A main effect of condition (*F*(2,46) = 12.03, *p* < .001, *ƞ*_p_^2^ = .34) and of value (*F*(1,23) = 4.57, *p* = .043, *ƞ*_p_^2^ = .17) was found. There was no statistically significant interaction effect (*p* > .05). No‐win trials (1.94 ± .39 μV) resulted in more positive potential amplitudes compared to both snatch (1.01 ± .28 μV; *p* < .001) and bargain trials (1.14 ± .30 μV; *p* = .001; see Figure 4e). Bargain and snatch trials did not significantly differ from each other (*p* > .05). High market value trials (1.21 ± .31 μV) resulted in a more positive potential amplitude than low market value trials (1.51 ± .31 μV, *p* = .043; see Figure 4e).

## 4 DISCUSSION

The present study shows for the first time that FRN and P300 can be elicited during the VA. Both FRN and P300 components differentiated between win and no‐win outcomes. Most notably, an FRN potential elicited at the vertex 251–271 ms post feedback‐onset differentiated between less favorable (snatch) and more favorable (bargain) wins—representing two extreme outcomes unique to the VA. In addition, the P300 amplitudes differentiated wins from no‐wins and between auction items of high and low retail price.

The production of an FRN demonstrates that VA bid outcomes were processed in a way comparable to outcomes in individual gambling tasks, such as a binary forced‐choice monetary gambling task (2AFC) (Gehring & Willoughby, 2002; Hajcak et al., 2007; Kokmotou et al., 2017; Yeung & Sanfey, 2004). Our findings support the involvement of a context‐dependent reward prediction error, as the FRN was primarily modulated by outcome valence (Holroyd et al., 2004; Holroyd & Coles, 2002). While the no‐win condition was objectively a financially neutral outcome, in the context of winning or “losing” an auction, it was the most unfavorable result.

The ability of the FRN to differentiate between the two types of win outcomes is also in line with reinforcement learning (Holroyd et al., 2004; Holroyd & Coles, 2002; Nieuwenhuis et al., 2004). The bargain win condition can be viewed as a reward of greater magnitude than the snatch win, as the difference between the participant's bid and the final price paid is larger. In the VA and BDM paradigms, one's bid value can also be referred to as a reservation price or indifference point, as paying one cent more than one's bid is a bad outcome (Padoa‐Schioppa, 2011). Therefore, the participants should be ambivalent toward a price outcome that is equal to their bid, and so the snatch condition is an intermediate outcome between the two extremes of bargain and no‐win. The greater FRN amplitude for bargain than snatch outcomes indicates that the FRN is sensitive to the relative value of a win (Holroyd et al., 2004; Meadows et al., 2016).

Violations of expectation may also have contributed to the difference in FRN amplitudes between snatch and bargain outcomes. The probability of each outcome is unknown in a VA task, unlike paradigms such as the 2AFC monetary gambling task, where participants are aware of the 50:50 chance of winning or losing (Gehring & Willoughby, 2002). The uncertainty caused by unknown outcome probabilities in the VA may have induced participants to rely on their own subjective values as an indicator of their opponent's behavior, and hence a predictor of likely outcomes. Correspondingly, the bargain condition would be considered a less probable win outcome as it indicates the misalignment of the participant's subjective value with that of their opponent. Therefore, the bargain result is the greater deviation from the expected reward magnitude (Bellebaum et al., 2010; Hauser et al., 2014).

In contrast with the previous study (Kokmotou et al., 2017), no correlations were found between any of the ERP components and loss aversion level. During a 2AFC monetary gambling task, loss aversion correlated positively with FRN amplitude at electrodes corresponding to the OFC, indicating a link between loss aversion implemented during risky decision‐making and a valuation process occurring in the OFC (Canessa et al., 2013). However, the associations between FRN and loss aversion seen in the study by Kokmotou and colleagues were based on FRN elicited during a task which involved real monetary losses in loss trials. Our findings suggest that the association between loss aversion and the FRN does not occur in the absence of a potential (monetary) loss. Therefore, the subjective framing of no‐wins as “losses” in an auction setting may be inadequate, and an objective risk of real loss is necessary to engage loss aversion mechanisms.

The P300 distinguished between no‐win and win outcomes, and between high and low market value results. However, the parameters of the study limits interpretation of the win versus no‐win amplitude differences due to the win outcomes being twice as frequent as the no‐win outcomes. As the P300 is well established to be sensitive to outcome probability (Polich, 2007, 2012), it cannot be ruled out that this difference impacted the observed win versus no‐win amplitudes.

As the P300 is involved in discerning motivational significance of outcomes (Bradley, 2009; Hajcak & Foti, 2020; Pfabigan et al., 2019; Wang et al., 2015), the attentional engagement and cognitive effort shown in auctions may be mediated by the market value of the item being auctioned (Meadows et al., 2016; Tyson‐Carr et al., 2018). This is also in‐line with the broader motivational significance framework (Bradley, 2009). As bid values were linked to market value, participants may have been more invested in the outcomes of items that they appreciated were worth more. This tendency would echo the sunk cost effect, where emotional and cognitive effort is extended in situations of financial commitment (Zeng, Zhang, et al., 2013; Zeng, Zou, & Zhang, 2013b). This would suggests that P300 component was sensitive to retail value as items of a higher retail price are more salient and engaging.

The present study was not without its limitations. Previous work has shown significant relationships between cortical activation changes during initial valuation of products and subsequent purchase decisions (Goto et al., 2017; Schaefer et al., 2016). As the pre‐bid period during the VA consists of free viewing of a displayed item, electrophysiological explorations would require recording and analysis of eye‐movement related potentials, similar to Tyson‐Carr et al. (2020), which was beyond the scope of the present study. A monetary threshold effect may have also impacted the results. As all wins are considered a good economic outcome, the degree of difference between the final price paid and one's bid could be of minor importance. Meanwhile, the social reward of beating an opponent brings another dimension to the outcome, and so “snatching” a win could be perceived as the “better” reward outcome (Chen, 2011).

This interplay of social and financial reward processing is a limitation of the present study, but could be unpacked by directly comparing a VA to a BDM to isolate the effect of a social dimension on reward processing mechanisms. Previous behavioral data have shown that, relative to a BDM, participant bidding behavior during a VA is more varied and divergent from the economically dominant strategy (Flynn et al., 2016). Further, fMRI studies using first‐price auctions have found emotional cue factors, such as risk aversion and loss contemplation, result in higher levels of overbidding and the winner's curse (Delgado et al., 2008; van den Bos et al., 2013). A comparison of the two mechanisms could be valuable for evaluating individual differences in replying on emotional cues during bidding.

Present data provides an initial insight into neural mechanisms underlying evaluation of decision outcomes in VA. Results show that receiving bid outcome information during a VA elicited an FRN potential at a latency and location that were compatible with FRN activity seen in other decision‐making tasks. The amplitude of the FRN also differentiated the favorability of VA win outcomes, a specific feature not seen in other demand‐revealing mechanisms. The VA also elicited a P300 component that encoded saliency related to the economic value of the items. Separation of value‐ and auction‐specific cortical responses provides important insight into decision‐making processes. Future exploration of the dynamics of Vickrey auctions has the potential for significant contributions to understanding the cognitive and neural systems that support economic decision‐making.

## AUTHOR CONTRIBUTIONS

**Alice Newton‐Fenner:** Conceptualization, data curation, formal analysis, investigation, methodology, project administration, writing – original draft. **John Tyson‐Carr:** Data curation; software; validation; writing – review and editing. **Hannah Roberts:** Investigation; methodology; writing – review and editing. **Jessica Henderson:** Data curation; methodology; writing – review and editing. **Danielle Hewitt:** Data curation; methodology; writing – review and editing. **Adam Byrne:** Methodology; writing – review and editing. **Nicholas Fallon:** Conceptualization; supervision; writing – review and editing. **Yiquan Gu:** Conceptualization; supervision; writing – original draft. **Olga Gorelkina:** Conceptualization; supervision; writing – review and editing. **Yuxin Xie:** Software; validation; writing – review and editing. **Athanasios Pantelous:** Supervision; writing – review and editing. **Timo Giesbrecht:** Conceptualization; funding acquisition; supervision. **Andrej Stancak:** Conceptualization; formal analysis; funding acquisition; supervision; writing – review and editing.

## FUNDING INFORMATION

This work was supported by the EPSRC and ESRC Centre for Doctoral Training [Grant Number: EP/L015927/1] on the Quantification and Management of Risk and Uncertainty in Complex Systems and Environments, University of Liverpool, Liverpool, UK, and Unilever.

## CONFLICT OF INTEREST

The authors declare that they have no conflict of interest.
